# Evaluation of the Corneal Endothelium Following Cataract Surgery in Diabetic and Non-Diabetic Patients

**DOI:** 10.3390/diagnostics13061115

**Published:** 2023-03-15

**Authors:** Adela-Laura Ciorba, George Roiu, Amir Mohamed Abdelhamid, Sameh Saber, Simona Cavalu

**Affiliations:** 1 Faculty of Medicine and Pharmacy, University of Oradea, 410087 Oradea, Romania; 2 Department of Pharmacology, Faculty of Pharmacy, Delta University for Science and Technology, Gamasa 7730103, Egypt

**Keywords:** diabetes, cataract surgery, phacoemulsification, corneal endothelium, cell density

## Abstract

The aim of this study was to evaluate the influence of phacoemulsification cataract surgery on the state of the corneal endothelium in diabetic versus non-diabetic patients. We compared the corneal cell morphology in 48 diabetics with good glycemic control and 72 non-diabetic patients before and after uneventful phacoemulsification. Corneal cell density, central corneal thickness, and hexagonality were measured preoperatively and post-surgery (at 1 and 4 weeks) by specular microscopy. The effect of age, gender, axial length, and anterior chamber depth on the parameters of the corneal endothelium were evaluated. We noticed a drop in the endothelial density in both groups postoperatively: a mean endothelial cell loss of 472.7 ± 369.1 in the diabetic group was recorded versus 165.7 ± 214.6 mean loss in the non-diabetic group after the first week. A significant increase in central corneal thickness was also noticed in both groups one week after phacoemulsification, but no statistical significance after 4 weeks in the diabetic group. In terms of cell hexagonality, statistically significant differences were noticed after 4 weeks in both groups. Overall, a significant difference between diabetic and non-diabetic population was noticed in terms of corneal endothelial cell loss after uneventful phacoemulsification cataract surgery. Routine specular microscopy and HbA1c evaluation is recommended before cataract surgery, while intraoperative precautions and high monitorisation in terms of pacho power intensity and ultrasound energy, along with a proper application of the dispersive viscoelastic substances are essential to reduce the risk of endothelial damage.

## 1. Introduction

In 2020, the leading cause of blindness all over the world was cataract, which affected 15.2 million people, among other pathologies such as uncorrected refractive error, glaucoma, age-related macular degeneration, and diabetic retinopathy [1]. Aging is the most common cause of cataract development (senile cataracts over the age of 65), but other factors, such as traumatic injuries (concussion or perforation), systemic diseases, endocrine disorders (diabetes, hypoparathyroidism), radiation, chemicals, or local diseases, such as uveitis and retinal detachment, can also cause cataract formation [2,3]. It is well known that the ability of corneal self-repair occurs within a mechanism characterized by enlargement of the endothelial cells. However, the posterior corneal surface is maintained by a gradual increase in the size of the remaining healthy cells, which result in increased cellular pleomorphism accompanied by a decrease in the percentage of hexagonal cells with age [4,5,6,7]. Any injury that causes loss or damage to the corneal endothelial cells may threaten vision, including traumatism or intraocular surgery [8]. Moreover, the presence of diabetes in elderly patients undergoing cataract surgery makes them even more vulnerable to greater endothelial damage, a hypothesis that has been demonstrated by several studies [9,10,11,12,13]. They are five times more likely than non-diabetic to develop cataract at an early age. One of the mechanisms responsible for the pathogenesis of cataract among diabetics seems to be the polyol pathway, through which the enzyme aldose reductase (AR) catalyses the reduction of glucose into sorbitol [14]. Diabetic patients with poor glycemic control have been found to have pleomorphism and polymegethism, which can be translated into diabetic corneal endotheliopathy [15,16]. The avascular cornea is very sensitive to prolonged hyperglycaemia, and, therefore, many diabetic patients develop corneal complications, such as recurrent erosions, delayed epithelial wound healing, sensitivity loss, or tear film alterations [17,18]. Anterior capsular phimosis is more common in diabetic eye patients, and, therefore, a larger capsulorhexis should be performed, but at the same time it should be smaller than the optic diameter to avoid another possible postoperative complication, posterior capsular opacification [15,16]. As a consequence, the endothelium of diabetic patients is more susceptible to trauma associated with surgery, as they possess a smaller pupil. Due to these aspects, there is a lack of room for handling the intraocular instruments, and a potential damage to the cornea is more likely to occur. Hence, difficulties in dilation and maintaining it with mydriatics are often mainly due to autonomic neuropathy, which predominantly involves the sympathetic innervation of the iris dilator [10]. Phacoemulsification surgery with intraocular lens (IOL) implantation is a newer and appealing technique for surgeons because it allows a faster and safer surgical procedure which involves smaller incisions for lens nucleus removing. The visual recovery time is shortened and the intraoperative complications, such as expulsive haemorrhage or suture-induced astigmatism are fewer compared to conventional extracapsular cataract extraction [19,20]. Fragmentation and emulsification of the lens involves the use of high-intensity ultrasound energy during phacoemulsification, which can damage the corneal endothelium due to elevated localised temperature or a prolonged time of phacoemulsification [21]. Upon applying the phacoemulsification technique, two main heat sources are involved: (1) heat due to the conversion of electrical energy into mechanical energy and (2) heat due to friction when the phaco needle vibrates against the sleeve that contains the probe. The thermal damage to the collagen fibres may occur when the temperature reaches 60 °C, but continuous irrigation applied during surgery has a cooling effect by preventing the contracture of the incision site and surrounding tissue [22,23,24,25,26]. Specular microscopy is used for in vivo analyzation of the corneal endothelium, being an excellent non-contact and non-invasive tool used for assessing the health of the corneal endothelium before and after surgery [27]. This allows a direct investigation of the morphology, distribution, and density of the endothelial cells, supporting the surgeon’s decision either that it is safe or to not proceed with the surgery, as the minimal cell density required for maintaining corneal transparency and not developing irreversible corneal oedema or bullous keratopathy is 700 cells/mm^2^ [28,29]. The average value for cell density in a healthy adult is 2400 cells/mm^2^; if there is a coefficient of variation above 0.40 or a hexagonality percentage below 50 it might not tolerate intraocular surgery [27]. It has an extremely valuable diagnostic role for different corneal dystrophies, such as Fuchs endothelial dystrophy, iridocorneal endothelial syndrome, or posterior polymorphous dystrophy [30]. In this context, we present our results related to the effect of phacoemulsification cataract surgery on the morphology of corneal endothelium in diabetic versus non-diabetic patients, aiming to evaluate the influence of age, gender, axial length, and anterior chamber depth on the characteristic parameters of the corneal endothelium.

## 2. Materials and Methods

This cross-sectional, retrospective study was conducted on 120 eyes from 120 patients in order to compare the corneal endothelium alterations in terms of corneal cell density (CD), central corneal thickness (CCT), and hexagonality (HEX) of the cells of 48 diabetic patients with a 10-year maximum history of the disease and good glycemic control (HbA1c < 7%) compared to 72 non-diabetic patients, before and after uneventful phacoemulsification surgery (1 and 4 weeks post-surgery) in the ophthalmology department of Emergency County Hospital Oradea, Bihor County, Romania. The study was conducted in tenets of the Declaration of Helsinki. Ethical approval was obtained from the Institutional Review Board of the hospital (no. 4238/11.02.2021).

### 2.1. Pre-Operatory Ophthalmological Examination

**Figure 1:**
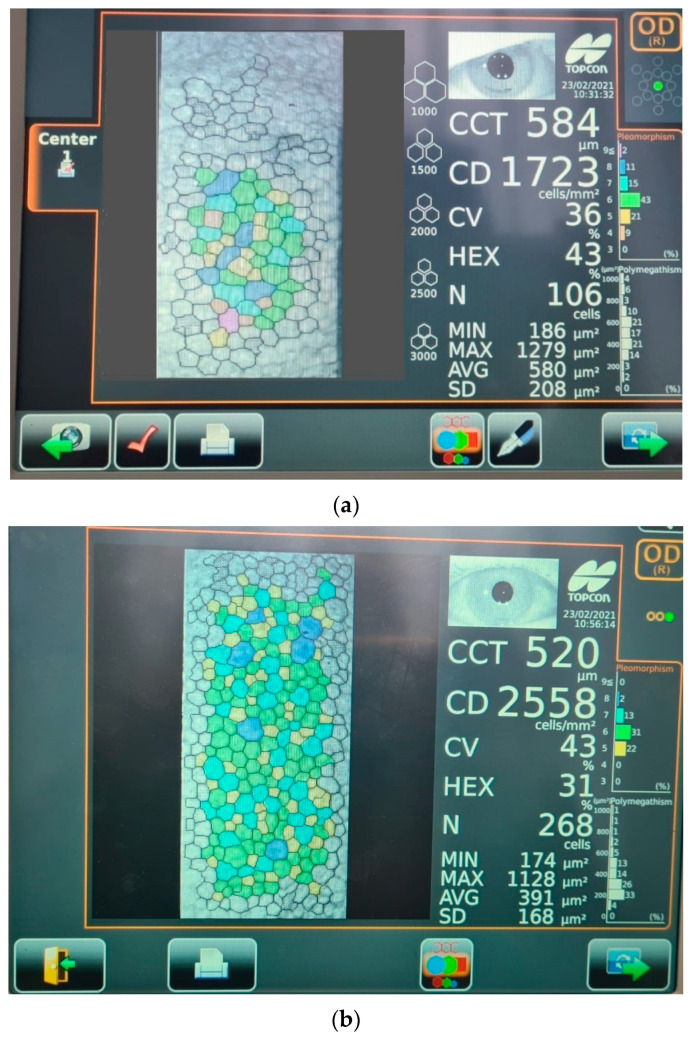
Representative images of endothelial cell layer (coloured dots) 1 week after surgery performed with specular microscopy for (**a**) diabetic patient and (**b**) non-diabetic patient. Legend: CCT—corneal central thickness; CD—cell density; CV—coefficient of variation; HEX—cell hexagonality; N—normal value; MIN—minimum value; MAX—maximum value; AVG—average value; SD—standard deviation of specular image; Pleomorphism—percentage of cells with variation from normal hexagonal shape; Polymegathism—size variation in the endothelial monolayer.

All patients underwent a rigorous ocular examination including visual acuity, slit-lamp examination, intraocular pressure using Goldman applanation tonometry, and a dilated fundus examination using a 90D lens. Corneal endothelial cell density (CD), central corneal thickness (CCT), and percentage of hexagonality (HEX) were measured both preoperatively and after surgery (at 1 and 4 weeks) using a noncontact specular microscope (Topcon Specular Microscope, Topcon Corporation Itabashi-ku, Tokyo, Japan 2017). A representative photograph recorded with the specular microscope showing the features of a non-diabetic cornea 1 week after surgery is displayed in Figure 1. The axial length (AXL) and the anterior chamber depth (ACD) were noted before surgery for each patient with immersion A-scan biometry, while the ultrasound energy consumption of the phaco machine and the effective phaco time were observed intraoperatively.

**Figure 2:**
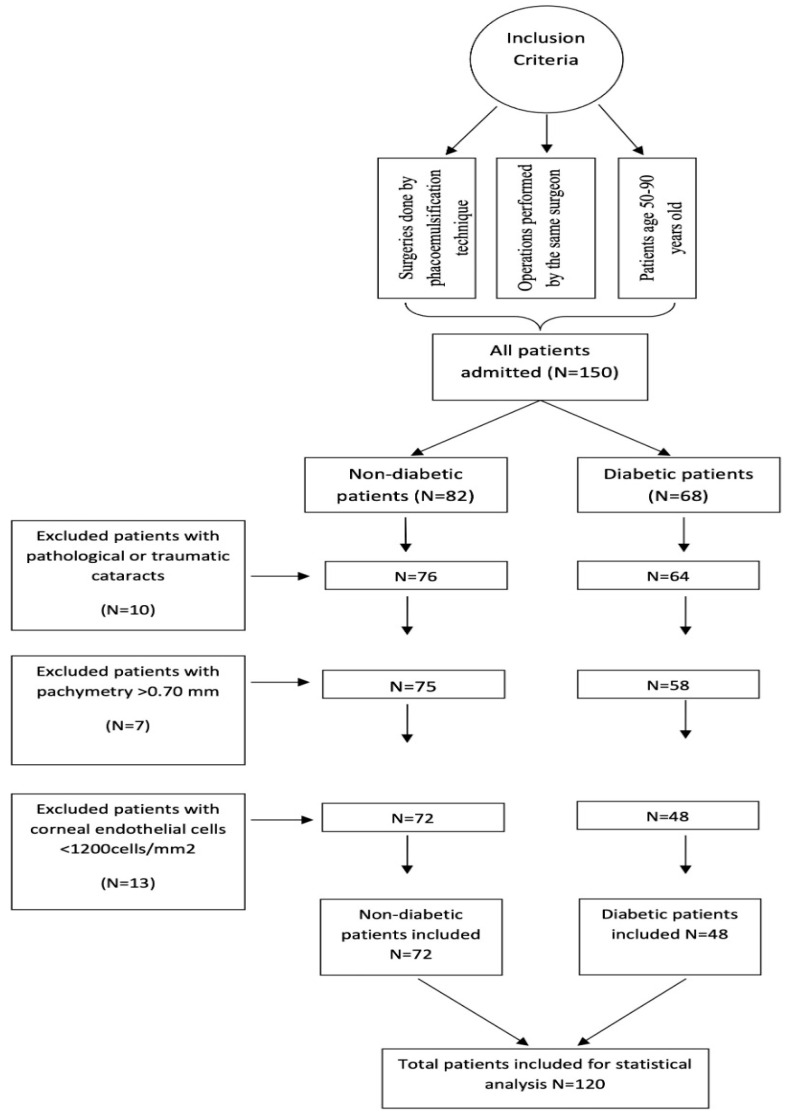
Study design inclusion and exclusion criteria chart.

The inclusion criteria of the study group consisted of patients with: (1) ages 50–90 years; (2) surgeries conducted by the same surgeon; and (3) phacoemulsification surgery technique, while the exclusion criteria were patients with: (1) pathological or traumatic cataracts; (2) pachymetry > 0.70 mm; and (3) corneal endothelial cells < 1200 cells/mm^2^ (Figure 2).

### 2.2. Surgical Technique

All surgical procedures were performed by an experienced surgeon (author G.R.) using the phacoemulsification machine (Stellaris PC, Bausch & Lomb, Bloomfield, CT, USA, 2016) with the “divide and conquer“ technique shifting into a “stop and chop”. Preoperatively, all the eyes were dilated using tropicamide 1% and phenylephrine 10%, followed by peribulbar anaesthesia. A main incision of 2.2 mm on the temporal side and two side-port incisions of 1.2 mm were applied at the limbus. A cohesive viscoelastic substance (Protectalon 2%) was injected into the anterior chamber for space maintenance, and a dispersive viscoelastic fluid was applied for corneal endothelium protection (Etacoat 2.4% HPMC—Hydroxypropyl Methyl Cellulose), then a curvilinear capsulorhexis was performed. Using phacoemulsification, the nucleus lens was removed along with the residual cortex, while irrigation–aspiration was applied. Different foldable IOLs (intraocular lenses), such as hydrophobic monofocal IOLs with a biconvex aspheric optic that ensures that the lens is virtually aberration neutral (Hyflex) or trifocal hydrophobic lenses (Acriva Trinova) were implanted in the capsular bag. The excess of viscoelastic materials was washed out and the corneal incisions were hydro-sealed with a special patch.

### 2.3. Statistical Analysis

Statistical analysis was carried out using GraphPad Prism (version 9.3.1). Numerical variables are presented as the mean ± standard deviation (SD). The correlations between different parametric variables before the operation were analyzed using Pearson correlation test. Repeated measure ANOVA following Tukey’s multiple comparisons test was used to compare the variables in the pre- and postoperative periods in one group. Unpaired *t*-test was used to compare the difference between diabetic and non-diabetic group. Correlation analysis was used to assess the univariate associations of the quantitative variables with endothelial cell loss. Multiple regression analysis was used to determine which variables independently contributed to the amount of endothelial cell loss. Significance was accepted at (*p* < 0.05).

## 3. Results

**Figure 3:**
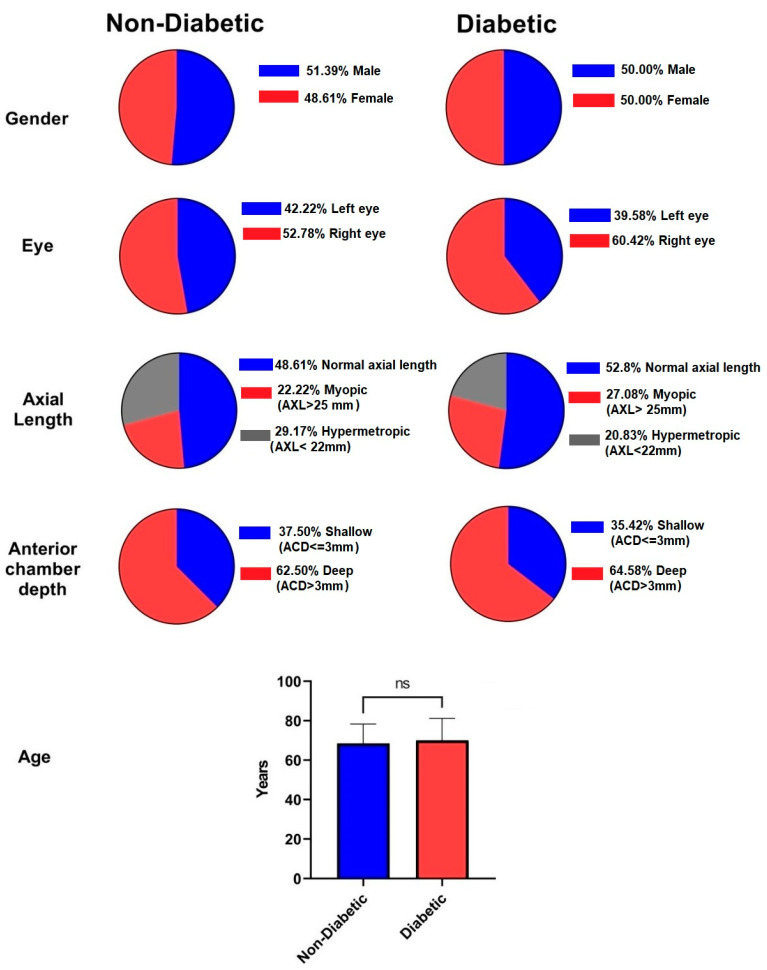
Combined socio-demographic and clinical characteristic of diabetic and non-diabetic groups. Legend: AXL—axial length; ACD—anterior chamber depth.

**Figure 4:**
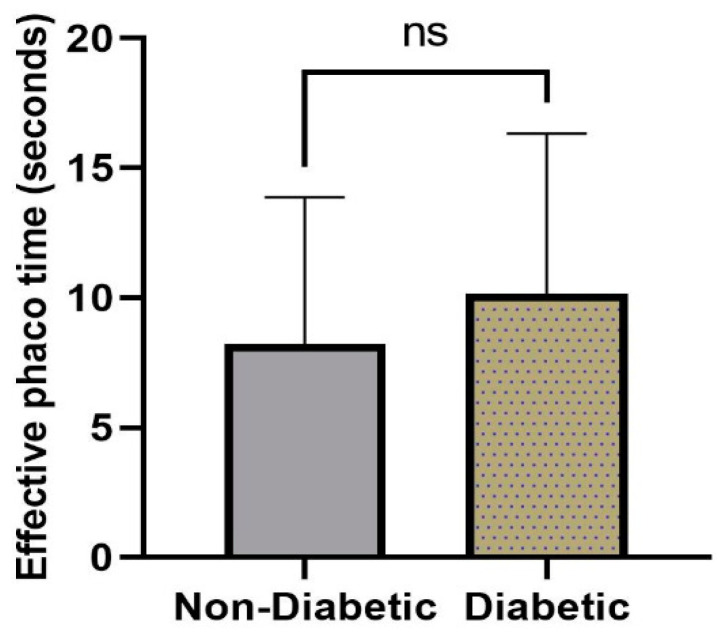
Effective phaco time (EPT) in diabetic versus non-diabetic group.

A total of 120 eyes were included in this study, respectively, 48 from diabetic and 72 from non-diabetic patients; the socio-demographic characteristics are presented in Figure 3. The mean age of the study population in the diabetic group was 70.06 ± 11.08 while in the non-diabetic group it was 68.65 ± 9.71 years, ranging from 48–88 years. The ratio between men and women in the diabetic group was 1:1, while in the non-diabetic group a slightly higher percent of males was noted. Based on the records, in the non-diabetic group, 22.22% of the patients presented with values of axial length (AXL) above 25 mm and 29.2% under 22 mm, while in the diabetic group, 27.1% of patients presented with AXL values over 25 mm and 20.8% under 22 mm. The anterior chamber depth (ACD) was over 3 mm in about 60% of both groups and under 3 mm in 30% of the groups. Of the operated eyes, 67 were right and 53 were left eyes; the clinical features of each are also summarized in Figure 3. There was no statistically significant difference between the two groups in terms of age or gender. Intraoperatively, the mean effective phaco time in the diabetic group was 10.15 ± 6.18 s, while in the non-diabetic group it was 8.23 ± 5.64 s, with a *p*-value of 0.0814 and no statistical difference between groups (Figure 4).

**Figure 5:**
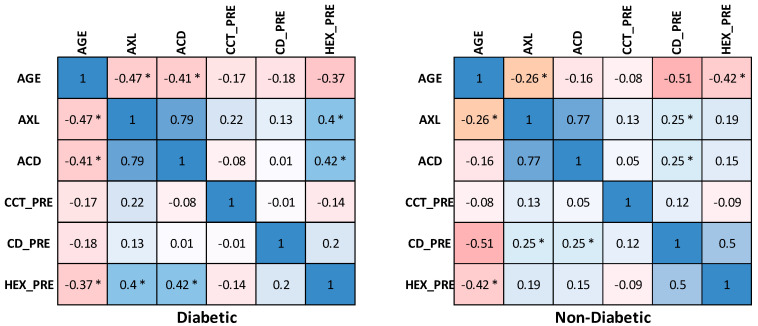
Correlation matrix between measured variables before the surgery. Legend: AXL, axial length; ACD, anterior chamber depth; CCT_PRE, central corneal thickness preoperative; CD_PRE, cell density preoperative; HEX_PRE, hexagonality of endothelial cells preoperative; * significance, *p* < 0.05.

The correlation matrix between different variables before surgery reveals that in the diabetic group, there was a moderate negative correlation between age and AXL, ACD, and HEX, whereas in the non-diabetic group, age showed a moderate negative correlation with CD and HEX. The HEX also had a moderate positive correlation with ACD and AXL in the diabetic group and a moderate positive correlation with CD in non-diabetics. Also, a very strong positive correlation between AXL and ACD was noticed in both diabetic and non-diabetic groups (Figure 5).

### 3.1. Corneal Endothelial Cell Count

**Figure 6:**
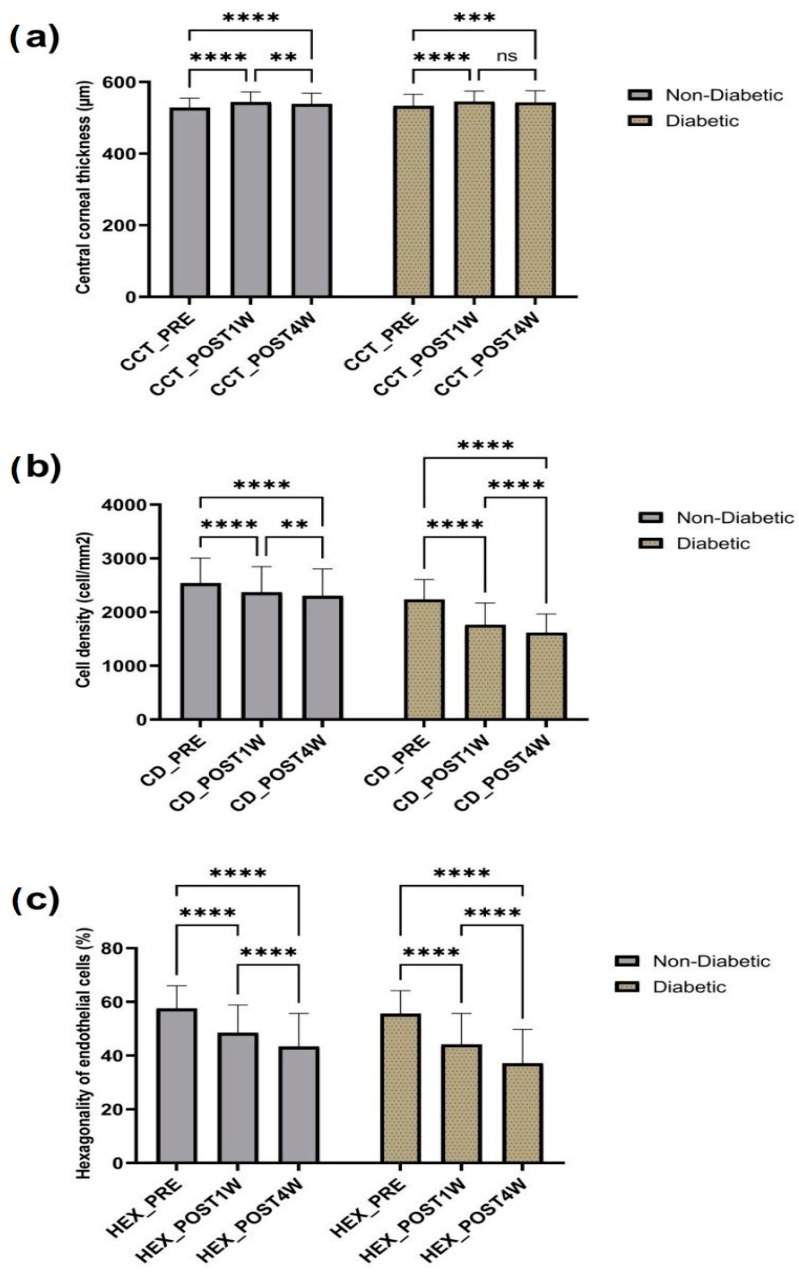
Effect of surgery on central corneal thickness (**a**), cell density (**b**), and hexagonality of endothelial cells (**c**). Data are presented as the mean ± SD and compared using repeated ANOVA. Legend: ns, non-significant, ** *p* < 0.01, *** *p* < 0.001, **** *p* < 0.0001. Legend: CCT, central corneal thickness; CCT_PRE—central corneal thickness preoperative; CCT_POST1W—central corneal thickness 1 week postoperative; CCT_POST4W—central corneal thickness 4 weeks postoperative; CD, cell density; CD_PRE—cell density preoperative; CD_POST1W—cell density 1 week postoperative; CD_POST4W-cell density 4 weeks postoperative; HEX, hexagonality; HEX_PRE—hexagonality preoperative; HEX_POST1W—hexagonality 1 week postoperative; HEX_POST4W—hexagonality 4 weeks postoperative.

The mean preoperative cell density (CD) was 2238 ± 369.6 in the diabetic group and 2537 ± 469 in the non-diabetic group. Comparing the postoperative cell loss in the non-diabetic group versus the diabetic group, a significantly higher endothelial cell loss was noted in the diabetic group after the first-week postoperative measurements (Table 1). More precisely, one week after the surgery the mean value of endothelial cell loss was 472.7 ± 369.1 in the diabetic group compared to 165.7 ± 214.6 in the non-diabetic group. It is also important to underscore the fact that diabetic patients lost more endothelial cells, progressively, from the 1st week post-surgical visit to the 4th week follow-up evaluation compared to their non-diabetic counterparts (Figure 6). 

### 3.2. Central Corneal Thickness (CCT)

The central corneal thickness increased in both groups after surgery, with a greater statistical significance in the first week post phacoemulsification (Figure 6). There was no statistical difference when the results of the non-diabetic and the diabetic group were compared by applying the unpaired *t*-test.

### 3.3. Percentage of Hexagonal Cells (HEX)

**Figure 7:**
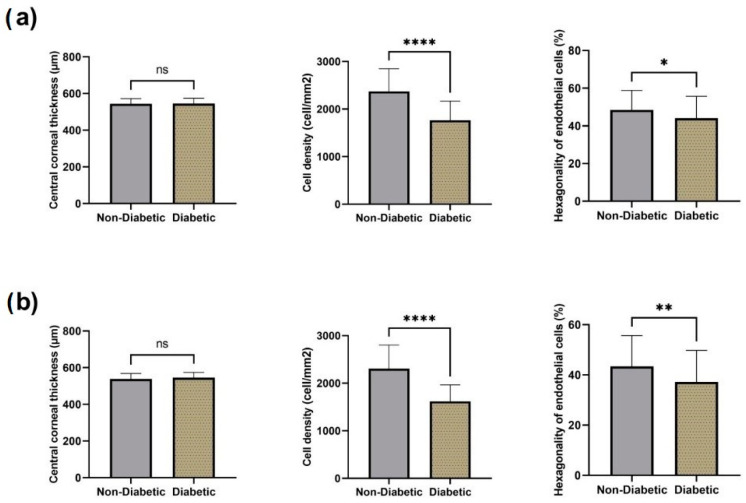
Effect of diabetes on surgical outcomes, including central corneal thickness, cell density, and hexagonality of endothelial cells postoperative at 1st week (**a**) and at 4th week (**b**). Data are presented as mean ± SD and compared using unpaired *t*-test. Legend: ns—non-significant, * *p* < 0.05, ** *p* < 0.01, **** *p* < 0.0001.

The percentage of hexagonal cells dropped in both groups after surgery. The first week after surgery a statistically significant difference (*p* < 0.05) between non-diabetic and diabetic groups was noted, with a greater difference observed at week 4 (*p* < 0.01) (Figure 7).

Corneal cells do not possess the ability to replicate, and hence, when their number decreases, a compensatory mechanism is initiated, causing enlargement of the remaining cells and loss of their hexagonal shape [28].

### 3.4. Univariate Analysis

By applying the univariate analysis, it could be observed that only preoperative cells density was significantly associated with endothelial cells loss in the diabetic group. On the contrary, the univariate analysis showed that no variable had any influence on the endothelial cell loss in the non-diabetic group (Table 2).

### 3.5. Multiple Regression Analysis

The analysis by multiple regression model was applied to find the best predictors of endothelial cell loss. The final model explained 22% of the variation in endothelial cell loss. The final model identified that being diabetic was an independent predictor of endothelial cell loss, while gender, age, ultrasound energy (U/S), axial length (AXL), anterior chamber depth (ACD) and effective phaco time were not independently associated with endothelial cell loss (Table 3).

## 4. Discussion

In the present study, the influence of phacoemulsification cataract surgery on the morphology of corneal endothelium was evaluated in diabetic versus non-diabetic patients. Our results revealed a reduction in the number of endothelial cells, highlighting the fact that cataract surgery is a traumatic procedure for the cornea, regardless of the presence or absence of diabetes. We also noticed an increase in the central corneal thickness in both groups after surgery, with a greater statistical significance in the first week post phacoemulsification, while the percentage of hexagonal cells dropped in both groups after surgery, with a greater difference observed at week 4 (*p* < 0.01).

High endothelial cell loss after phacoemulsification was described as being related to age, ocular comorbidities such as cornea guttata or other corneal dystrophies [31], ocular trauma, anterior chamber depth [32], eyes with occludable angles, or the inexperience of surgeons [33]. The patients with diabetes seem to be more prone to endothelium damage. In our study, after phacoemulsification, a decrease in the number of endothelial cells was noticed in each group individually, but comparatively, the cells loss in the diabetic group was statistically more significant than in the non-diabetic group (a mean endothelial cell loss of 472.7 ± 369.1 versus 165.7 ± 214.6). Similar to our study, the research conducted by Yang et al. [34] and Maadane et al. [35] reported significant corneal endothelial cell loss after cataract surgery, both in the diabetic group and the control group. Sharma et al. [36] also obtained similar results: the CD was lower in the diabetic patients than in the control group, respectively, 2550 ± 326 versus 2634 ± 256, but no difference was noted in the mean pachymetry or hexagonality. Similar results regarding the postoperative CD values were also found in Muhammad Khalid’s study [37]. They included 80 patients with type 2 diabetes and 80 patients without diabetes, with the results showing CD loss of 14.88% (±10.79) in the diabetic group and 9.86% (±10.20) in the control group (*p* = 0.003). The study conducted by Lee and Choi showed a mean CCT that was significantly higher in the diabetic group (588 ± 2.7 µm) and also a lower cell density compared to the control group (2577.2 ± 27.3 cell/mm^2^ vs. 2699.9 ± 38.7 cell/mm^2^) [38]. 

Amira and Shams [39] investigated 57 diabetic eyes and 45 non-diabetic eyes and found a lower endothelial cell density in the diabetic cornea group. A lower percentage of hexagonal cells (33.24% ± 10.25%) compared to that of the control group (34.24% ± 8.73%) was observed, but with no statistically significant difference (*p* = 0.603). Central corneal thickness was also noted to increase in the diabetic group after cataract surgery, with values of 545.61 ± 30.39 μm compared to 539.42 ± 29.22 μm in the control group, but it was not statistically significant (*p* = 0.301). 

A large study by Sudhir et al. on 1191 type 2 diabetic patients and 121 controls showed that in the diabetic group the endothelial cell count was lower when compared to the non-diabetic group, but no differences were found between the groups regarding the pachymetry values, hexagonality %, or coefficient of variation of cell size [36]. Leem et al. [40] reported that central corneal thickness was increased and endothelial cell density was decreased in patients with diabetes mellitus, and contact lens usage significantly affected corneal morphology in diabetic patients, although we did not include contact lenses users in our study. Xi Liu et al. evaluated the tear film of 25 diabetic patients and 20 non-diabetic patients after phacoemulsification cataract surgery. Alterations in the tear film and tear secretion were observed in both diabetic and non-diabetic patients after phacoemulsification, but with a greater reduction of the Schirmer I test in the diabetic group. The tear film break-up time values in both groups significantly decreased the first day after cataract surgery compared with the preoperative values, but when measured 180 days after phacoemulsification, the values returned to their preoperative ones in both groups [41]. Contrary, in a prospective study conducted by Storr-Paulsen et al. [6], the authors reported no significant differences between type 2 diabetic patients with good glycemic index and nondiabetic control subjects in terms of CD or hexagonality values [42]. 

Enlargement of endothelial cells, reduction of their hexagonality, and mobilisation of the remaining existing cells represent a mechanism of self-repair as a secondary effect to CD loss [6,40]. Many studies show that the corneal endothelial cells of diabetic patients have a decreased hexagonality and an increased coefficient of variation compared to those of non-diabetic patients [43,44,45], whereas others show no differences [42,46]. The greater sensitivity of the entire corneal endothelium of the diabetic patients may be explained by the accumulation of sugar alcohol in cells, which is converted by the excessive presence of glucose, as well by the fact that diabetic patients possess a reduced activity of Na/K-ATPase, which translates into structural instability and loss of the regular hexagonal pattern [47]. 

The endothelial cell loss was not significant enough in any of the patients included in our study, diabetic or non-diabetic, for postoperative complications to take place, such as corneal decompensation or bullous keratopathy. This is probably due to the use of viscoelastic substances that protect the corneal endothelium, careful use of ultrasound energy used for nucleus lens removal, a reduced effective phaco time, and also a good HbA1c (7%) in the diabetic group patients. However, as a general approach, the risk of corneal decompensation should always be the main concern in diabetic patients as the severity of the disease increases; additionally, in patients with diabetic retinopathy and nephropathy, extreme caution should be exercised.

There were a few limitations in our study. It was a cross-sectional, retrospective study, and hence, the measurements were made at a certain time point, and not all clinical variances could be precisely determined, while a prospective, controlled, and blinded design study would have allowed better quality interpretation of data. A single surgeon performed all surgeries, while with multiple surgeons we would probably have had a wider palette of postoperative results. Another limitation was the short-term follow-up of the study groups, although several studies showed that 4 weeks after uneventful cataract surgery is enough time for follow-ups, as the visual outcome at the end of 1 month is optimal and the postoperative complications usually occur in the first 2 weeks [48,49].

## 5. Conclusions

In our study, the influence of phacoemulsification cataract surgery on corneal cell density, central corneal thickness, and hexagonality was evaluated preoperatively and post-surgery (at 1 and 4 weeks) using specular microscopy. The effects of age, gender, axial length, and anterior chamber depth on the parameters of corneal endothelium of diabetic and non-diabetic patients were examined. We noticed significant differences between pre-surgical and postoperative CD values in both diabetic and non-diabetic patients. Despite good glycemic control, diabetic patients had more pronounced morphological abnormalities compared to those of non-diabetics, but visual outcomes after phacoemulsification with IOL implantation were similar in both groups. A drop in the postoperative endothelial density was noted after the first week, in both groups. A significant increase in central corneal thickness was also noted in both groups one week after phacoemulsification, but there was no statistical significance after 4 weeks in the diabetic group. In terms of cell hexagonality, statistically significant differences were noted after 4 weeks in both groups. 

A major finding in our study is that, although an advanced loss of CD was noted, along with an increased CCT and a reduction of hexagonality (especially in the diabetic group), there were no cases of postoperative bullous keratopathy, probably due to several factors, such as surgeon’s experience and the use of viscoelastic substances with a protective role, as well as a careful preoperative evaluation and a good glycemic index (HbA1c < 7%). 

We strongly recommend routine specular microscopy and HbA1c evaluation before all cataract surgeries. Regarding intraoperative precautions, a high level of monitoring is necessary in terms of pacho power intensity and ultrasound energy, along with a proper application of the dispersive viscoelastic substances to reduce the risk of endothelial damage for a successful surgical procedure.

## Figures and Tables

**Table 1:** Endothelial cell loss (cell/mm^2^) (mean ± SD).

	Diabetic Patients	Non-Diabetic Patients	*p*-Value (Diabetic vs. Non-Diabetic)
Preoperative CD	2238 ± 369.6	2537 ± 469	0.0003
1 week postoperative CD	1765 ± 403.1	2372 ± 476.7	<0.0001
Endothelial cell loss (n)	473 ± 369.1	165 ± 241.6	<0.0001
Endothelial cell loss (%)	21.13%	6.5%	

Legend: CD—cell density.

**Table 2:** Univariate association of the measured variables with the cell density loss.

Variable	Diabetic Patients		Non-Diabetic Patients	
	Correlation Coefficient (r)	*p* Value	Correlation Coefficient	*p* Value
Age	0.2381	0.1032	−0.1573	0.1871
AXL	−0.0191	0.8973	0.0962	0.4215
ACD	0.0147	0.921	0.1593	0.1814
CCT_PRE	−0.1020	0.4903	0.1993	0.0933
CD_PRE	0.4045	0.0044 **	0.1930	0.1044
HEX_PRE	0.0968	0.5128	−0.1171	0.3271
U/S	0.2841	0.0504	−0.1438	0.2282
EPT	0.1518	0.3032	0.0541	0.6515

Legend: AXL—axial length; ACD—anterior chamber depth; CCT_PRE—central corneal thickness preoperative; CD_PRE—cell density preoperative; HEX_PRE—hexagonality preoperative; U/S—ultrasound energy consumption; EPT—effective phase time; ** *p* < 0.01.

**Table 3:** Multiple regression model of the measured variables with the cell density loss.

Variables	Parameter Estimate Adjusted for All Other Listed Variables	*p* Value	Parameter Estimate (Final Model)	*p* Value
Gender [F]	93.31	0.0794	-	
DM [diabetic]	290.1	<0.0001 *	307	<0.0001 *
Age	1.146	0.6984	-	
AXL	−7.171	0.6410	-	
ACD	83.91	0.2901	-	
U/S (%)	247.0	0.7537	-	
EPT	4.83	0.4125	-	

Legend: AXL—axial length; ACD—anterior chamber depth; U/S—ultrasound energy consumption; EPT—effective phaco time. * *p* < 0.0001.

## Data Availability

The data supporting the reported results are available in the medical archive of Emergency County Hospital Oradea, Bihor, Romania.
